# Determining the optimal daily gonadotropin dose to maximize the oocyte yield in elective egg freezing cycles

**DOI:** 10.1186/s12958-024-01236-4

**Published:** 2024-06-06

**Authors:** Raoul Orvieto, Anouk Savir Kadmon, Nira Morag, Aliza Segev-Zahav, Ravit Nahum

**Affiliations:** 1 Department of Obstetrics and Gynecology, Chaim Sheba Medical Center, Ramat Gan, Israel; 2 Faculty of Medical and Health Science, Tel-Aviv University, Tel Aviv, Israel; 3 The Tarnesby-Tarnowski Chair for Family Planning and Fertility Regulation, Faculty of Medical and Health Science, Tel-Aviv University, Tel Aviv, Israel; 4 Arrow Program for Medical Education, Chaim Sheba Medical Center, Ramat Gan, Israel

**Keywords:** IVF, ovarian stimulation, Gonadotropin daily dose, Elective egg freezing

## Abstract

**Objective:**

Ovarian stimulation (OS) with high daily gonadotropin doses are commonly offered to patients attempting social/elective egg freezing. However, the optimal daily gonadotropin dose that would allow a higher oocyte yield in the successive IVF cycle attempt was not settled and should be determined.

**Patients and methods:**

Data from all women admitted to our IVF unit for social/EEF, who underwent two consecutive IVF cycle attempts, with only those who used in the first attempt a starting daily gonadotropin dose of 300IU were analyzed. Patients characteristics and OS variables were used in an attempt to build a logistic model, helping in determining the daily gonadotropin dose that should be offered to patient during their second EEF attempt, aiming to further increase their oocyte yield.

**Results:**

Three hundred and thirteen consecutive women undergoing two successive IVF cycle attempts were evaluated. Using logistic regression model, two equations were developed using individual patient-level data that determine the daily gonadotropin dose needed aiming to increase the oocyte yield in the successive cycle. (a): X=-0.514 + 2.87*A1 + 1.733*A2–0.194* (E2/1000) and (b): P = EXP(X) / [1 + EXP(X)].

**Conclusions:**

Using the aforementioned equations succeeded in determining the daily gonadotropin dose that might result in increasing oocyte yield, with an AUC of 0.85. Any additional oocyte retrieved to these EEF patients might get them closer to fulfil their desire to parenthood.

**Supplementary Information:**

The online version contains supplementary material available at 10.1186/s12958-024-01236-4.

## Introduction

Ovarian stimulation (OS) is considered a key factor in the success of in vitro fertilization-embryo transfer (IVF-ET), enabling the recruitment of multiple oocytes and, thereby, resulting in multiple embryos [1]. In 2019, the European Society for Human Reproduction and Embryology (ESHRE) special interest group (SIG) Reproductive Endocrinology [2] published has provided evidence-based information on the different options for OS for IVF/ICSI and concluded that a gonadotropin dose higher than 300 IU *is not recommended* for predicted poor responders.

What about normal, or high responder patients? Benadiva et al. [3] compared the hormonal profiles of ovulatory high and low responder patients who were treated with different daily doses (225 and 150 IU) of gonadotropins in successive IVF cycles. The magnitude of the ovarian response was found to be altered by changing the dose of gonadotropins in high responders, but not in normal responders. Moreover, while the increases in hormone levels accompanying a high response to gonadotropins could be dampened by lowering the dose, hormone concentrations were not influenced by changing the dose of gonadotropins in low responders.

Elective egg freezing (EEF) has spread globally following its endorsement by the American Society for Reproductive Medicine (ASRM) in 2018 [4]. In Israel, EEF for non-medical reasons is allowed for women age 30 to 41yrs. Moreover, while according to the Israel Health Law, almost every woman up to age 45 may legally seek *government-subsidized* infertility treatment with her own oocytes for as many cycles as necessary for the birth of two children, social/EEF for non-medical reason is not subsidized.

Ovarian stimulation (OS) with high daily gonadotropin doses, is therefore commonly offered to this group of patients, aiming to achieve the maximal oocytes cohort with minimum IVF cycle attempts. In a previous study of women undergoing EEF [5], the first OS stimulation attempt with high daily gonadotropin doses (300IU) yielded 8–9 oocytes, while the second attempt, using lower, same or increased daily gonadotropin dose, yielded an unpredictable increased, no change or decreased oocyte yield. In this study, it was therefore not possible to determine the optimal daily gonadotropin dose that would allow a higher oocyte yield in the successive IVF cycle attempt.

Prompted by the aforementioned observations, we aim to determine the daily gonadotropin dose in the second IVF cycle attempt, based on data retrieved from patients undergoing two successive attempts for EEF.

## Patients and methods

We reviewed the computerized files of all consecutive women admitted to our IVF unit, between February 2018 and March 2023, and reached the ovum pick-up (OPU) stage. The elimination of bias in this selection was achieved by including only women undergoing OS, using a starting daily gonadotropin dose of 300IU in the first IVF cycle attempt. The study was approved by our institutional review board (SMC-9589-22).

All women underwent the multiple dose GnRH-antagonist protocol with GnRH-agonist for triggering final follicular maturation, as previously described [5]. Data on patients’ characteristics and OS variables were collected from the files.

Results were presented as means + standard deviations. Differences in variables between the two cycles were statistically analyzed by student’s paired t-test and differences between the two doses, in the second cycle, were analyzed by the independent t-test. Multivariate analysis to estimate dose selection was performed by logistic regression models. The analyses included independent variables/covariates that were statistically significant in the univariate analyses. Level of significance was set at 0.05, and was two-tailed. The final logistic model equation was used to the estimate dose prediction.

## Results

Three hundred and thirteen consecutive women undergoing two successive IVF cycle attempts were evaluated. Women age and body mass index were 35.3 *±* 3.5yrs and 23.3 *±* 4.3 kg/m2, respectively. The IVF cycle characteristics are shown in Table 1. While there was no between- cycle difference in the duration of OS, peak E2 and progesterone levels and the number of follicles > 13 mm in diameter on the day of trigger, were significantly higher during the second IVF cycle attempt compared to the first attempt (Table 1). Moreover, during the second IVF cycle attempt women used a significantly higher daily gonadotropin dose (366 *±* 76 vs. 300 *±* 0; *p* < 0.001) and yielded more follicles *≥* 13 mm in diameter on day of trigger (9.7 *±* 5.0 vs. 8.6 *±* 4.1; *p* < 0.001), and more oocytes (12.37 *±* 6.5 vs. 10.5 *±* 5.9; *p* < 0.001) and mature oocytes (9.3 *±* 5.3 vs. 7.8 *±* 5.3; *p* < 0.001).

**Table 1:** IVF cycle characteristics

OS variables	1st IVF attempt	2nd IVF attempt	*p*-value
Daily Gonadotropin dose (IU) Duration of stimulation (days) Number of follicles > 13 mm in diameter Peak E2 (pmol/L) Peak progesterone (nmol/L) Number of oocytes retrieved Number of mature oocytes retrieved	300 ± 0 10.2 ± 1.9 8.6 ± 4.1 8806 ± 5574 2.7 ± 1.6 10.5 ± 5.9 7.8 ± 4.5	360 ± 76 10.4 ± 1.7 9.7 ± 5.0 9496 ± 6233 2.9 ± 1.9 12.3 ± 6.5 9.3 ± 5.3	< 0.001. NS < 0.001 < 0.04 < 0.04 < 0.001 < 0.001

OS- ovarian stimulation; NS- not significant.

An equation was developed using the logistic regression model for an individual patient-level data. This equation calculated the daily gonadotropin dose based on previous results including the oocyte yield in the first cycle.

### Step 1: Calculate X using the first equation (a)

X=-0.514 + 2.87*A1 + 1.733*A2–0.194* (E2/1000)

If the number of oocyte retrieved in the first cycle is *≤* 7: A1 = 1 and A2 = 0.

If the number of oocyte retrieved in the first cycle is between 8 and 12: A1 = 0 and A2 = 1.

And If the number of oocyte retrieved in the first cycle is *≥* 13, A1 = 0 and A2 = 0.

E2 is the estradiol level (pmol/L).

### Step 2: After calculating the X value, it should be placed in the following logistic model (b)

P = EXP(X) / [1 + EXP(X)].

If *P* > 0.5 then the suggested daily gonadotropin dose in the successive cycle should be 450IU, while if *P* < 0.5 it should be 300IU.

The correlation between the predicted and actual values was found to be good (AUC = 0.85).

*For example*, patient yielding 10 oocytes in the first cycle, using a daily gonadotropin dose of 300IU, with peak E2 of 8000 pmol/L.

Using equation (a):

X=-0.514 + 2.87*0 + 1.733*1–0.194* (8000/1000)= -0.33

Now, placing the X value in (b):

P = EXP(-0.333) / [1 + EXP(-0.333)] = 0.4175

Since P is < 0.5, the suggested daily gonadotropin dose should be 450IU.

*Calculator*: To simplify the calculation of the suggested daily FSH dose in the second IVF cycle attempt, a calculator is attached, where we have to place the # of oocytes retrieved and the peak E2 level in the first IVF cycle attempt, in the red and blue square, respectively.

## Discussion

In the present study, by analyzing the data of women undergoing two successive IVF cycle attempts for EEF, we could build a logistic model, helping in determining the daily gonadotropin dose that should be offered to patient during their second EEF attempt, aiming to further increase their oocyte yield. Since any improvement in patients’ response to OS, such as from low (1–3 oocytes) to suboptimal response (4–9 oocytes) was demonstrated to improve cumulative LBR [6], any additional oocyte retrieved to patients undergoing EEF might get them closer to parenthood.

As was previously demonstrated by Drakopoulos et al. [7], a dose increment of FSH remained the only significant predictor of the number of oocytes retrieved in the subsequent IVF cycle, with an increase of 50 IU of the initial rFSH dose leading to one more oocyte. Moreover, increasing the daily gonadotropin dose above 300IU was also shown to result in higher mature oocytes yield [5] in EEF patients. However, when analyzing the data according to the number of oocytes retrieved in the second IVF cycle attempt as compared to the first attempt [5], an increase, no change or reduced oocyte yield in the second cycle attempt was observed regardless the daily gonadotropin dose in the second cycle attempt (whether it was increased, no change and decreased compared to the first attempt).

In the present study, we managed to create equations, based on the OS variable in the first EEF cycle attempt, which helped determining the required daily gonadotropin dose that should be offered to EEF patients in their second IVF cycle attempt, in an attempt to increased their oocyte yield, with AUC of 0.85.

A major strength of our study is that we used and compared the IVF outcome in same cohort of “healthy” patients, undergoing two successive IVF cycle attempts for elective egg freezing (EEF). The fact that all women that participated in our study had two consecutive treatment cycles helps to eliminate multiple bias factors and to attribute the study results daily gonadotropin dose. On the other hand, biases might still exist either because of differences in time periods between the two cycles, statistical return to the mean, or other potential biases inherent in a retrospective analysis of the IVF cycles.

To conclude, patients attempting social/elective egg freezing has spread globally. While OS with high daily gonadotropin doses (300IU) should be offered to this group of patients in their first IVF cycle attempt, the daily gonadotropin dose in their second attempt aiming to achieve the maximal oocytes cohort with minimum IVF cycle attempts need to be determine. In the present study we offer a logistic model aiming to resolve this query. Further well designed prospective studies are needed to improve and validate our observation.

### Electronic supplementary material

Below is the link to the electronic supplementary material.

Supplementary Material 1

## Data Availability

No datasets were generated or analysed during the current study.
